# Effects of EPSPS Copy Number Variation (CNV) and Glyphosate Application on the Aromatic and Branched Chain Amino Acid Synthesis Pathways in *Amaranthus palmeri*

**DOI:** 10.3389/fpls.2017.01970

**Published:** 2017-11-16

**Authors:** Manuel Fernández-Escalada, Ainhoa Zulet-González, Miriam Gil-Monreal, Ana Zabalza, Karl Ravet, Todd Gaines, Mercedes Royuela

**Affiliations:** ^1^Departamento Ciencias del Medio Natural, Universidad Pública de Navarra, Pamplona, Spain; ^2^Department of Bioagricultural Sciences and Pest Management, Colorado State University, Fort Collins, CO, United States

**Keywords:** glyphosate, aromatic amino acid pathway, branched chain amino acid pathway, mRNA relative expression, EPSPS, CM, AS, *Amaranthus palmeri*

## Abstract

A key enzyme of the shikimate pathway, 5-enolpyruvylshikimate-3-phosphate synthase (EPSPS; EC 2.5.1.19), is the known target of the widely used herbicide glyphosate. Glyphosate resistance in *Amaranthus palmeri*, one of the most troublesome weeds in agriculture, has evolved through increased *EPSPS* gene copy number. The aim of this work was to study the pleiotropic effects of (*i*) *EPSPS* increased transcript abundance due to gene copy number variation (CNV) and of (*ii*) glyphosate application on the aromatic amino acid (AAA) and branched chain amino acid (BCAA) synthesis pathways. Hydroponically grown glyphosate sensitive (GS) and glyphosate resistant (GR) plants were treated with glyphosate 3 days after treatment. In absence of glyphosate treatment, high *EPSPS* gene copy number had only a subtle effect on transcriptional regulation of AAA and BCAA pathway genes. In contrast, glyphosate treatment provoked a general accumulation of the transcripts corresponding to genes of the AAA pathway leading to synthesis of chorismate in both GS and GR. After chorismate, anthranilate synthase transcript abundance was higher while chorismate mutase transcription showed a small decrease in GR and remained stable in GS, suggesting a regulatory branch point in the pathway that favors synthesis toward tryptophan over phenylalanine and tyrosine after glyphosate treatment. This was confirmed by studying enzyme activities *in vitro* and amino acid analysis. Importantly, this upregulation was glyphosate dose dependent and was observed similarly in both GS and GR populations. Glyphosate treatment also had a slight effect on the expression of BCAA genes but no general effect on the pathway could be observed. Taken together, our observations suggest that the high CNV of *EPSPS* in *A. palmeri* GR populations has no major pleiotropic effect on the expression of AAA biosynthetic genes, even in response to glyphosate treatment. This finding supports the idea that the fitness cost associated with *EPSPS* CNV in *A. palmeri* may be limited.

## Introduction

**FIGURE 1:**
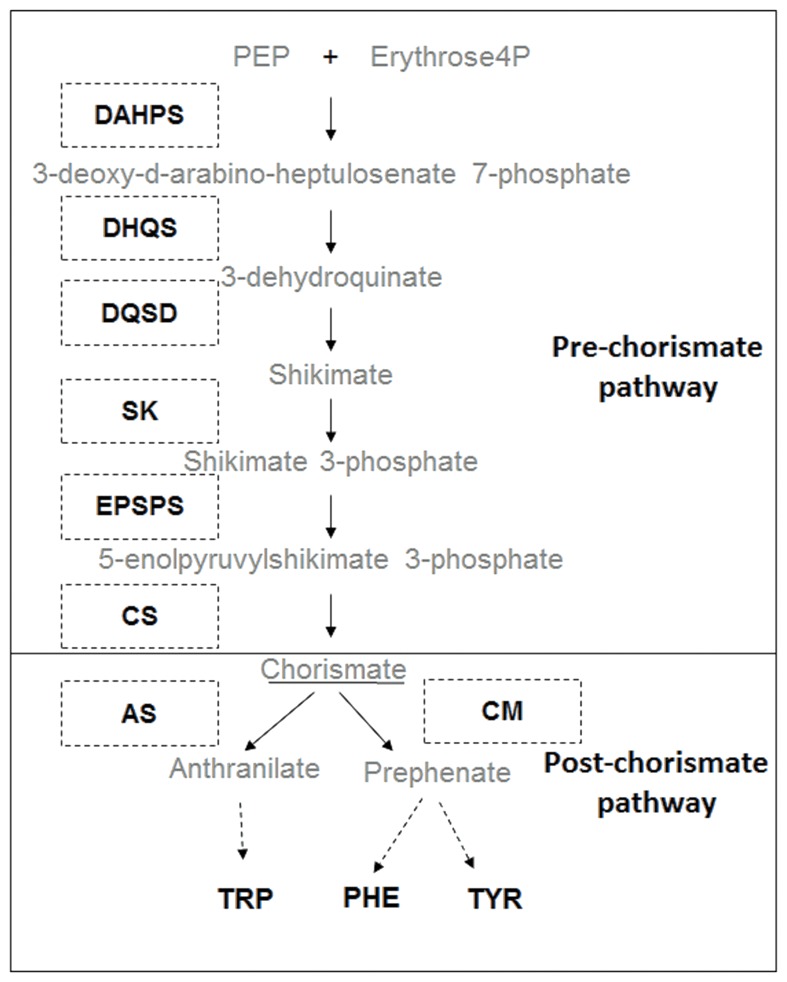
Biosynthetic pathway of aromatic amino acids (AAAs). Consecutive enzymatic steps of pre-chorismate pathway: D-arabino-heptulosonate 7-phosphate synthase (DAHPS), dehydroquinate synthase (DHQS), 3-dehydroquinate dehydratase/shikimate dehydrogenase (DQSD), shikimate kinase (SK), 5-enolypyruvylshikimate 3-phosphate synthase (EPSPS), and chorismate synthase (CS); and post-chorismate pathway: anthranilate synthase (AS) chorismate mutase (CM) leading to the synthesis of tyrosine (TYR), phenylalanine (PHE), and tryptophan (TRP).

The shikimate pathway uses carbon from primary metabolism to form chorismate, a precursor of the essential aromatic amino acids (AAAs) phenylalanine (Phe), tyrosine (Tyr), and tryptophan (Trp) (Tzin and Galili, 2010). These AAAs are not only essential components of protein synthesis but also serve as precursors for a wide range of secondary metabolites with multiple biological functions in plants, including plant stress tolerance (Dyer et al., 1989; Keith et al., 1991; Gorlach et al., 1995; Janzik et al., 2005; Maeda and Dudareva, 2012). The AAA synthesis pathway can be subdivided into two steps: (i) the pre-chorismate (shikimate) pathway which provides the precursor chorismate used for synthesis of all AAAs and (ii) the post-chorismate pathway which can lead to either synthesis of Phe and Tyr, or Trp, via different routes (**Figure 1**) (Maeda and Dudareva, 2012). Synthesis of chorismate is catalyzed by seven enzymes acting sequentially (**Figure 1**): D-arabino-heptulosonate 7-phosphate synthase (DAHPS), dehydroquinate synthase (DHQS), 3-dehydroquinate dehydratase/shikimate dehydrogenase (DQSD), shikimate kinase (SK), 5-enolypyruvylshikimate 3-phosphate synthase (EPSPS), and chorismate synthase (CS). After formation of chorismate, synthesis of Trp is catalyzed by anthranilate synthase (AS) while synthesis of Phe and Tyr is catalyzed by chorismate mutase (CM) (Tohge et al., 2013).

Due to its importance for plant biology, the synthesis of AAA is a tightly regulated process controlled by many inputs (Bentley and Haslam, 1990; Tzin and Galili, 2010; Tohge et al., 2013; Galili et al., 2016). Four points appear as checkpoints: the entrance of the pathway with the enzyme DAHPS (Sato et al., 2006), an exit of major importance with the phenylalanine ammonia lyase (PAL) (Hahlbrock and Scheel, 1989), the branch point in the post-chorismate pathway (Maeda and Dudareva, 2012) and the enzyme EPSPS. The enzyme EPSPS is the target of the herbicide glyphosate (Steinrücken and Amrhein, 1980) and therefore a key step in the shikimate pathway.

The intensive and continuous use of glyphosate has led to the emergence of glyphosate resistant (GR) weed populations (Powles, 2008). The global issue of herbicide resistance for weed management is a serious challenge for global food security (Délye et al., 2013). One of the most damaging glyphosate-resistant weed species is *Amaranthus palmeri* S. Wats (Culpepper et al., 2006; Powles and Yu, 2010). Glyphosate resistance is conferred by gene amplification of *EPSPS*, which leads to a massive production of the enzyme EPSPS (Gaines et al., 2010). The recommended field dose is not sufficient to inhibit EPSPS activity, and plants survive. Copy number variation (CNV) of *EPSPS* is now reported to confer glyphosate resistance in several weed species including *Lolium multiflorum* (Salas et al., 2012) and *Kochia scoparia* (Wiersma et al., 2015) and particularly in *Amaranthus* species such as *Amaranthus tuberculatus* (Lorentz et al., 2014) and *Amaranthus spinosus* (Nandula et al., 2014).

To date, how the AAA pathway is regulated and how glyphosate may affect this regulation is not clearly understood. In particular, it is unknown whether there are pleiotropic effects associated with *EPSPS* CNV, particularly at the AAA synthesis pathway. Notably, no fitness cost has been associated with massive increase of EPSPS activity in GR populations (Giacomini et al., 2014; Vila-Aiub et al., 2014). However, the gene amplification resistance mechanism found in *A. palmeri* offers us the opportunity to study the regulation of the shikimate pathway, the effect of *EPSPS* overexpression due to extra *EPSPS* gene copies, and the effect of glyphosate application. In addition to the feedback regulation of AAA biosynthetic pathway, the hypothesis of the existence of cross-regulation of amino acid metabolic pathways at the transcriptional level has been revised (Pratelli and Pilot, 2014). A close correlation between AAA and branched chain amino acids (BCAAs) has been found (Noctor et al., 2002).

In this study, the main objective was to evaluate the impact of *EPSPS* overexpression by gene amplification and of glyphosate treatment on the regulation of the AAA pathway and free AAA content. To this aim, the response of glyphosate sensitive (GS) and GR populations of *A. palmeri* to glyphosate were evaluated at the molecular and biochemical levels. Additionally, *m*RNA relative expression of the main enzymes from the BCAA pathway was developed to test whether there is any variation in their levels because of the overexpression of *EPSPS* or glyphosate treatment.

## Materials and Methods

### Plant Material and Herbicide Application

Seeds of *A. palmeri* GS and GR biotypes were originally collected from North Carolina (United States) (Chandi et al., 2012; Fernández-Escalada et al., 2016). The resistance mechanism of the GR biotype has been described to be *EPSPS* gene amplification (Chandi et al., 2012), with 47.5 more gene copies in GR than in GS plants (Fernández-Escalada et al., 2016).

Plants were germinated and grown in aerated hydroponic culture under controlled conditions according to procedures described in Fernández-Escalada et al. (2016). Three week-old plants [after reaching the growth stage defined as BBCH 14 (Hess et al., 1997)] were treated with glyphosate (commercial formula, Glyfos, 360 g a.e. L^-1^, isopropylamine salt, BayerGarden, Valencia, Spain) at both recommended field rate (1 × = 0.84 kg ha^-1^) and three times that rate (3 × = 2.52 kg ha^-1^), according to Culpepper et al. (2006). Glyphosate treatment was performed using an aerograph (Junior Start model; Definik; Sagola). Control plants were treated with water. At 3 days after treatment, leaves were collected, frozen, and ground to a fine power as previously described (Fernández-Escalada et al., 2016). The experiment was conducted twice.

### Quantitative Reverse Transcription-PCR

RNA was extracted from leaf tissues using the Machery-Nagel NucleoSpin^®^ RNA Plant kit following manufacturer’s instructions. Total RNA concentration was measured with Gen 5.1.11 (Biotek Instruments, Inc., United States) and RNA quality was assessed using RNA gel electrophoresis. The gels were visualized using a Gel Doc 2000 system (BIORAD Laboratories, Inc., Hercules, CA, United States).

cDNA synthesis was performed using BIORAD iScript^TM^cDNA Synthesis Kit with 1 μg of total RNA following manufacturer’s instructions.

Quantitative RT-PCR (qRT-PCR) was performed using a Thermocycler BIORAD CFX Connect TM Real-Time System. The reaction kit used for qPCR was PerfeCTa SYBR^®^ Green SuperMix (Quantabio, Beverly, MA, United States). Each reaction was performed using 1 μL of cDNA template. The following thermal profile was used for all PCRs: denaturation at 95°C for 2 min, 40 cycles of 95°C for 15 s and 52–61°C for annealing and extension for 20 s. Optimal annealing temperature for each primer was determined using gradient PCR. All primers and annealing temperatures are listed in Supplementary Table 1. *EPSPS* primer was modified from Gaines et al. (2010). Melting curve analysis was conducted to verify amplification of single PCR products. Gene expression was monitored in five biological replicates. Primer efficiency (E) for each primer is presented in Supplementary Table 1 and was calculated according to E = 10[-1/slope] (Pfaffl, 2001). Relative transcript level was calculated as E_GOI_^CP_GOI control_-CP_GOI treated_^/E_REF_^CP_REF control_-CP_REF treated_^ (Pfaffl, 2001), where GOI = gene of interest, REF = reference gene (beta tubulin was used as normalization gene), and CP = crossing point, the cycle at which fluorescence from amplification exceeded the background fluorescence. Relative transcript level was calculated for all genes of the AAA synthesis pathway, corresponding to eight enzymes and four genes of the BCAA synthesis pathway.

### EPSPS, DAHPS, and PAL Immunoblotting

Protein extraction was performed using 0.1 g of ground leaf tissue in 0.2 mL of extraction buffer (MOPS 100 mM, EDTA 5 mM, Triton-X 100 1%, glycerin 10%, KCl 50 mM, benzamidine 1 mM, iodoacetamide 100 μM, PVP 5% and PMSF 1 mM). Proteins were separated by 12.5% SDS-PAGE and immunoblots were produced according to standard techniques. The protein amount loaded per well for each antibody used is specified in the figure legends. EPSPS and DAHPS antibody dilutions were 1:2000 (Fernández-Escalada et al., 2016) and 1:1000 (Orcaray et al., 2011), respectively. PAL antibody was produced by a custom peptide facility (Biogenes, Berlin, Germany) using a short, conjugated peptide as an antigen (C-QFAKPR-SDSFEEKN). The antibody was raised in rabbits using standard protocols from the manufacturer, and the primary antibody dilution was 1:500. An anti-rabbit AP conjugated antibody (Sigma Chemical, Co., St. Louis, MO, United States) was used as a secondary antibody at a dilution of 1:20000. Bands were identified using a BCIP/NBT kit which was Amplified alkaline phosphatase immunoblot assay kit (BIORAD 170, BIORAD Laboratories, Inc., Hercules, CA, United States). Immunoblots were scanned using a GS-800 densitometer, and protein bands were quantified using Quantity One software (BIORAD Laboratories, Inc., Hercules, CA, United States). In the case of EPSPS protein, membrane signals were normalized according to total soluble protein loading quantity. In the case of DAHPS and PAL, absolute signals were used.

### Enzymatic Activities

5-Enolpyruvylshikimate-3-phosphate synthase activity was performed using the procedure described in Gaines et al. (2010). PAL activity was carried out according to Orcaray et al. (2011) with the following modifications. Samples were immediately centrifuged after extraction (12,000 *g*, 5 min) The reaction was started by the addition of 25 mM L-phenylalanine (Maroli et al., 2015). Controls (without L-phenylalanine) were prepared to determine endogenous levels of transcinnamic acid (t-CA). Incubation was performed for 1 h at 37°C (Sarma et al., 1998; Wang et al., 2007).

Protein extraction for CM and AS activity assays was developed as described in Singh and Widholm (1974) with addition of 1 mM PMSF (Goers and Jensen, 1984). Samples were desalted using PD-10 columns (Ishimoto et al., 2010). CM enzymatic activity was measured as described in Goers and Jensen (1984). Control for each sample was carried out using enzymatic extracts previously inactivated with 1 N HCl. AS activity was quantified as described in Ishimoto et al. (2010). Controls were performed using boiled enzymatic extract (Matsukawa et al., 2002).

### Shikimate Determination

For shikimate content determination, three leaf disks (4 mm diameter) were excised from the youngest leaf of each plant. Leaf disks were placed in a screw-top 2 mL Eppendorf tube, frozen, and stored at -80°C until analysis. Shikimate was extracted as described in Koger et al. (2005). After addition of 100 μL of 0.25 N HCl per disk to each vial, samples were incubated at 22°C for 1.5 h and mixed by vortexing. Shikimate content was quantified spectrophotometrically (Cromartie and Polge, 2000).

### Aromatic Amino Acid Content Determination

Ground leaf (0.1 g) was homogenized in 1 M HCl for amino acid extraction. Protein precipitation was performed after incubation on ice and centrifugation (Orcaray et al., 2010). After derivatization with fluorescein isothiocyanate, AAA content was measured by capillary electrophoresis coupled to a laser-induced fluorescence detector, as described in Zulet et al. (2013b). Analyses were performed at 20°C and at a voltage of +30 kV. For tryptophan determination, the voltage was reduced to +20 kV in order to improve separation.

### Statistical Analysis

Transcript level analyses were performed using five biological replicates. For immunoblot, enzyme activity, shikimate and AAA quantification, four biological replicates were used. One-way ANOVA with a multiple-comparison adjustment for least significant difference (LSD) at *p* ≤ 0.05 was used. Statistical analyses were performed using SPSS Statistics 24.0 (IBM, Corp., Armonk, NY, United States).

## Results

**FIGURE 2:**
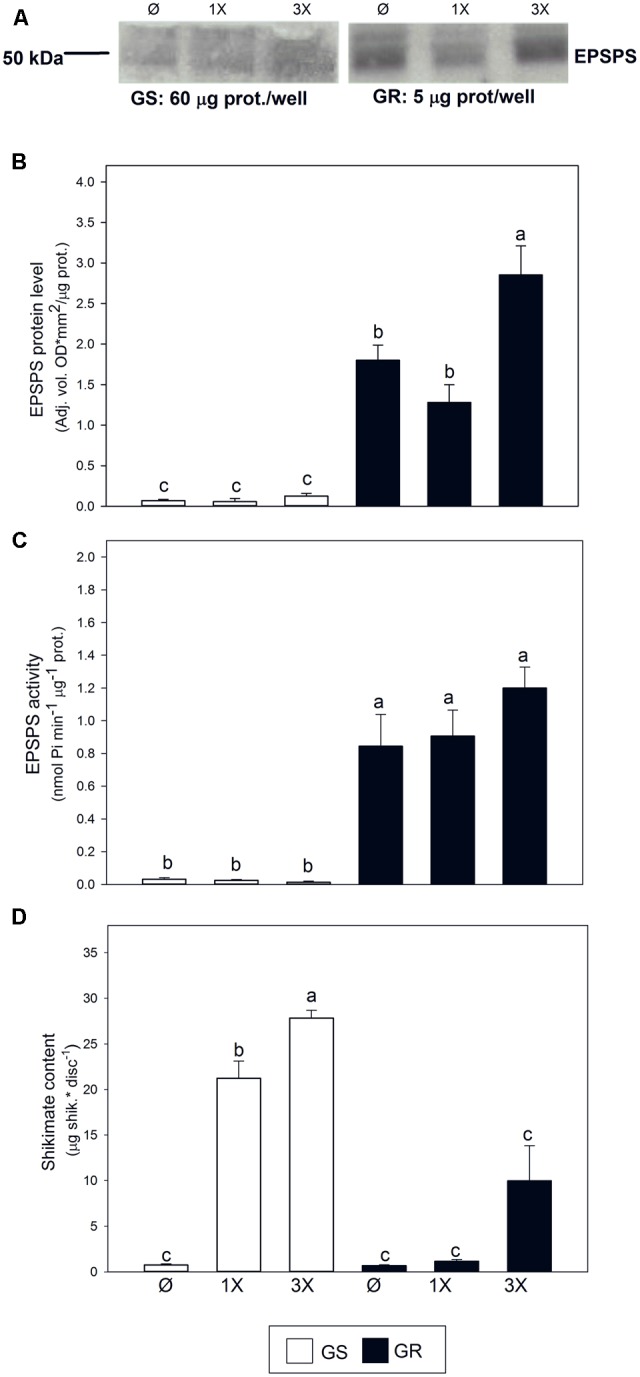
Characterization of resistance in *Amaranthus palmeri* populations. Glyphosate sensitive (white bars; GS) and glyphosate resistant (black bars; GR) populations were untreated (Ø) or treated with glyphosate and measured 3 days after treatment with one (1X) or three times (3X) field dose. **(A)** Representative immunoblots for EPSPS. Total soluble proteins (60 μg for GS or 5 μg for GR) were fractioned by 12.5% SDS-PAGE and blotted. **(B)** Normalization of the intensity of the EPSPS bands expressed as optical density for unit of area per μg of protein (Mean ± SE; *n* = 3). **(C)** EPSPS *in vitro* enzymatic activity measured spectrophotometrically in semicrude leaf extracts (Mean ± SE; *n* = 4). **(D)** Shikimate content was measured spectrophotometrically after extraction from leaf disks of treated plants (Mean ± SE; *n* = 4). Different letters indicate significant differences between treatments and/or populations (*p*-value ≤ 0.05, LSD test).

The number of *EPSPS* copies in the studied GR biotype was reported to be 47.5 fold when compared to the corresponding GS biotype (Fernández-Escalada et al., 2016). In the absence of glyphosate, protein level was increased by 25 fold (**Figures 2A,B**) and EPSPS activity was 26 fold higher (**Figure 2C**). In response to glyphosate, only a mild increase of the abundance of EPSPS protein was observed in the GR biotype at the highest glyphosate dose (**Figures 2A,B**). EPSPS activity was not affected by glyphosate in the GR biotype, regardless of the dose, while it was slightly decreased in the GS biotype with the highest dose applied (**Figure 2C**). While shikimate content was almost negligible in untreated plants of both populations, it accumulated after glyphosate treatment in GS and in GR only at the highest glyphosate dose. Shikimate accumulated significantly more in GS than in GR at each glyphosate dose (**Figure 2D**), confirming the inhibition of EPSPS by glyphosate observed in GS (**Figure 2C**).

**FIGURE 3:**
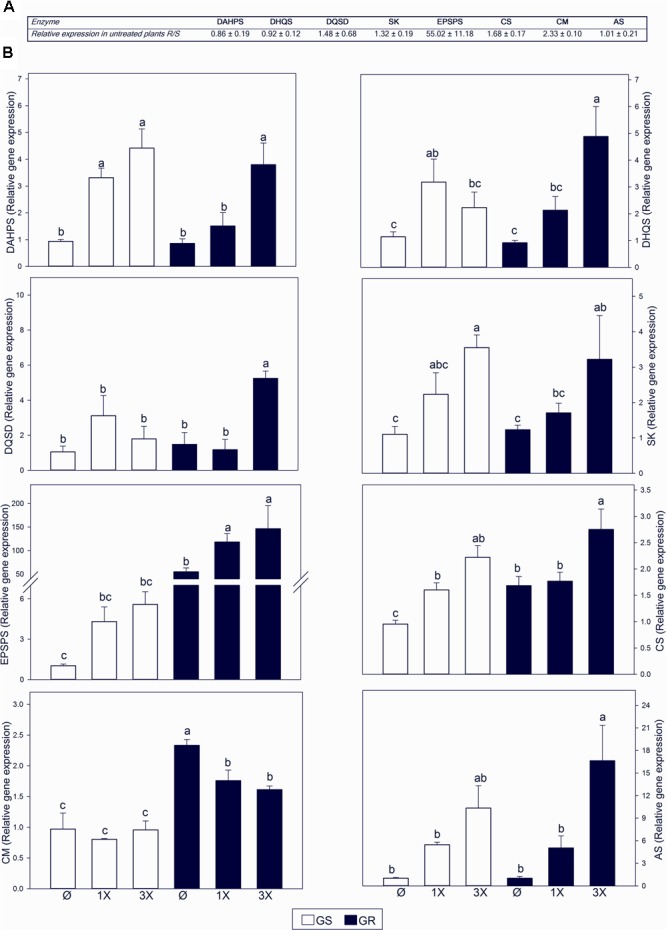
Transcript abundance of genes in the aromatic amino acid (AAA) biosynthetic pathway. Glyphosate sensitive (white bars; GS) and glyphosate resistant (black bars; GR) populations were untreated (Ø) or 3 days after treatment with glyphosate at one (1X) or three times (3X) field dose. Enzyme abbreviations as described in **Figure 1**. **(A)** Ratio of GR to GS relative transcript abundance measured with qRT-PCR normalized using the normalization gene *beta tubulin*. **(B)** Relative transcript abundance normalized using the normalization gene *beta tubulin*, relative to GS untreated plants (Mean ± SE; *n* = 5). Different letters indicate significant differences between treatments and/or populations (*p*-value ≤ 0.05, LSD test).

To study the impact of the high *EPSPS* copy number on the regulation of the AAA biosynthetic pathway, transcript levels for seven enzymes were analyzed by qRT-PCR. In absence of glyphosate treatment, *EPSPS* transcript level was increased by 55 fold in GR (**Figure 3A**), confirming the results of Fernández-Escalada et al. (2016). For other enzymes, particularly *CS* and *CM*, only marginal changes were observed (1.68 and 2.33 fold, respectively) (**Figure 3A**).

Glyphosate provoked an induction of the expression of all the genes of the shikimate pathway, with the exception of *CM* (**Figure 3B**). The change in gene expression was dose dependent. The same effect was observed in both GS and GR populations. *CM* showed the opposite behavior, with no change (GS) or a slight decrease (GR) in *CM* transcript accumulation after treatment with glyphosate (**Figure 3B**). The most responsive gene was *AS* with upregulation over 15 fold in GR with the highest dose (**Figure 3B**). This may suggest a preferential flux to the Trp biosynthesis branch rather than to the Phe and Tyr branch in response to glyphosate treatment.

**FIGURE 4:**
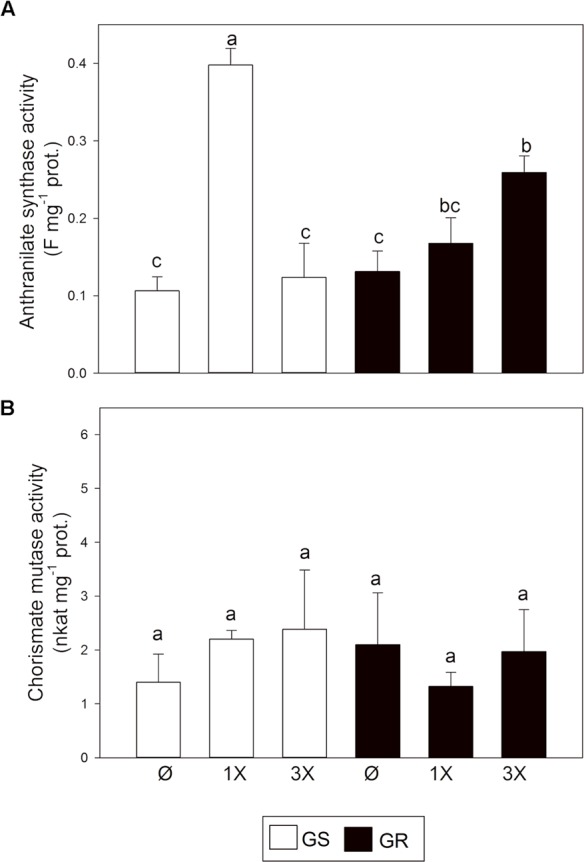
Anthranilate synthase (AS) and chorismate mutase (CM) enzymatic activities. Glyphosate sensitive (white bars; GS) and glyphosate resistant (black bars; GR) populations were untreated (Ø) or 3 days after treatment with glyphosate at one (1X) or three times (3X) field dose. **(A)** AS was measured in desalted leaf extracts by measuring the fluorescence of the produced anthranilate. **(B)** CM was measured in desalted leaf extracts by measuring prephenate production spectrophotometrically (Mean ± SE; *n* = 4). Different letters indicate significant differences between treatments and/or populations (*p*-value ≤ 0.05, LSD test).

To pursue this hypothesis, the activity of CM and AS enzymes was studied. In the absence of glyphosate, AS (**Figure 4A**) and CM (**Figure 4B**) activities were similar in both biotypes. Changes in the activity of AS and CM confirmed the trend observed at the transcript level, suggesting a preferential synthesis toward Trp after glyphosate treatment. *AS* expression induction was concomitant with an increase in the enzyme activity while CM activity was unchanged.

**FIGURE 5:**
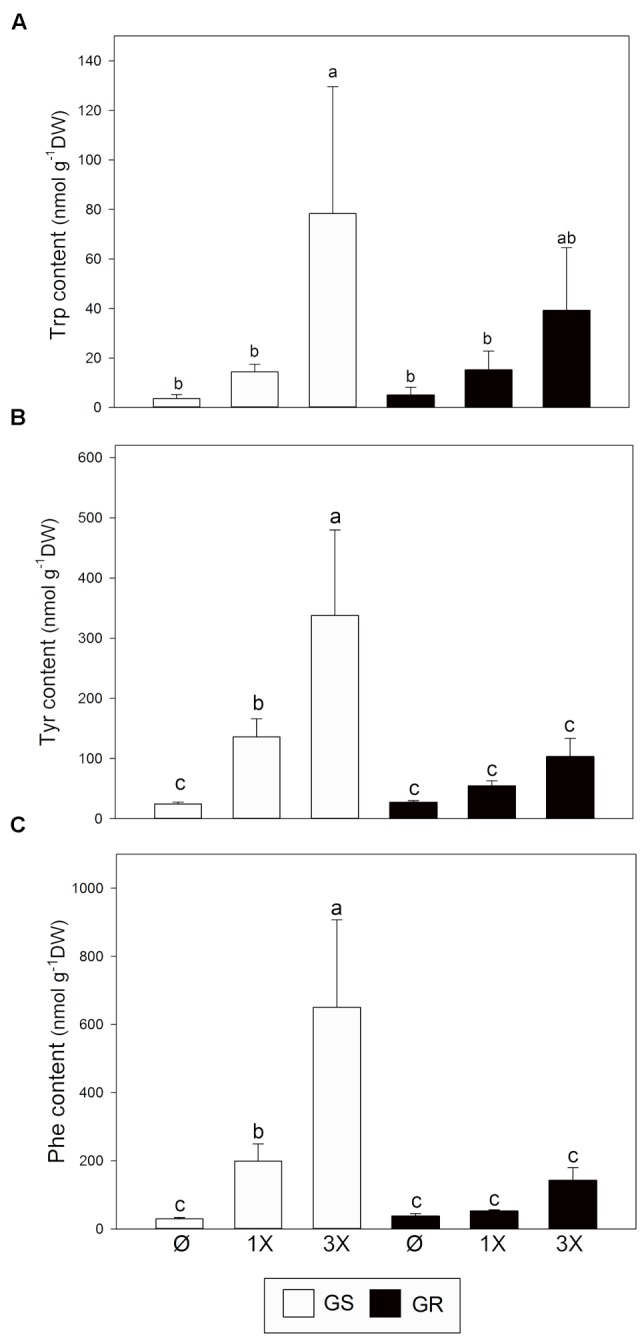
Aromatic amino acid content. Glyphosate sensitive (white bars; GS) and glyphosate resistant (black bars; GR) populations were untreated (Ø) or 3 days after treatment with glyphosate at one (1X) or three times (3X) field dose. Tryptophan (Trp; **A**), tyrosine (Tyr; **B**), and phenylalanine (Phe; **C**) were measured by capillary electrophoresis in leaf acidic extracts (Mean ± SE; *n* = 4). Different letters indicate significant differences between treatments and/or populations (*p*-value ≤ 0.05, LSD test).

Next, AAA levels were measured (**Figure 5**). Before treatment with glyphosate, levels of Trp (**Figure 5A**), Tyr (**Figure 5B**), and Phe (**Figure 5C**) were similar in both GS and GR biotypes. This result confirms that the striking change in *EPSPS* expression due to CNV does not have a major effect on AAA levels. After glyphosate treatment, the level of all AAA increased (**Figures 5A–C**). However, significant changes were detected only in GS. In GR, the highest increase was detected for Trp.

**FIGURE 6:**
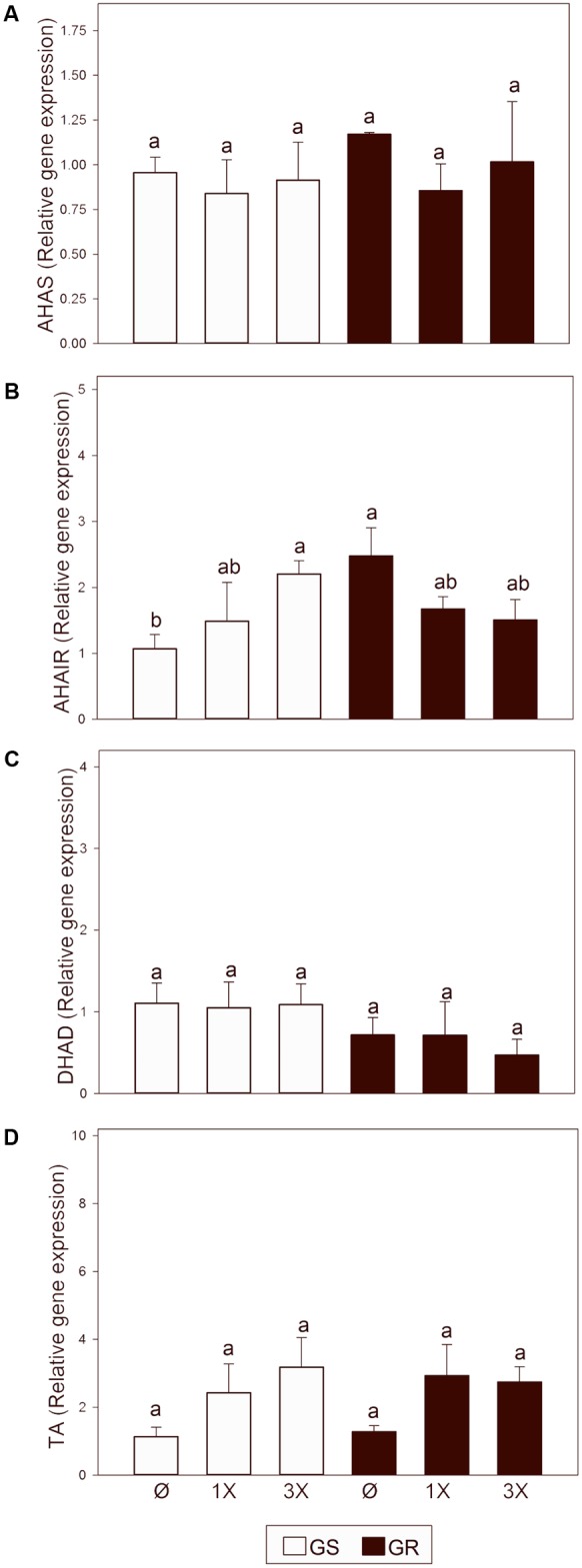
Transcript abundance of genes in the branched chain amino acid (BCAA) biosynthetic pathway. Glyphosate sensitive (white bars; GS) and glyphosate resistant (black bars; GR) populations were untreated (Ø) or 3 days after treatment with glyphosate at one (1X) or three times (3X) field dose. Relative expression of acetohydroxyacid synthase (AHAS; **A**) ketol-acid reductoisomerase (AHAIR; **B**), dihydroxyacid dehydratase (DHAD; **C**) and branched-chain amino acid transaminase (TA; **D**) normalized with the normalization gene *beta tubulin*, and relative to untreated GS plants (Mean ± SE; *n* = 5). Different letters indicate significant differences between treatments and/or populations (*p*-value ≤ 0.05, LSD test).

Previous studies with the same populations and the same time of study and concentration of glyphosate provoked a threefold increase of total free amino acid content and a 12 fold increase of BCAA content (Fernández-Escalada et al., 2016). The higher effect of glyphosate on BCAA content than on other amino acid types suggests a possible effect of the herbicide on the BCAA biosynthetic pathway. Based on this, the expression pattern of four enzymes of BCAA biosynthetic pathway was also measured: acetohydroxyacid synthase (AHAS), ketol-acid reductoisomerase (AHAIR), dihydroxyacid dehydratase (DHAD) and branched-chain amino acid transaminase (TA) (**Figure 6**). Transcript abundance of the BCAA biosynthetic pathway was not different between the untreated plants of both populations, suggesting that *EPSPS* overexpression does not affect BCAA pathway expression. After glyphosate treatment, *AHAS, DHAD*, and *TA* showed no change at either dose in GS or in GR. *AHAIR* transcript abundance was increased in GS at the highest glyphosate dose, while it did not change in GR after glyphosate treatment.

## Discussion

### Characterization of Resistance in *A. palmeri* Populations

In the GR population of *A. palmeri* an *EPSPS* gene amplification (Fernández-Escalada et al., 2016) results in a massive increase of the accumulation of corresponding transcript (**Figure 3A**) and of the protein level and activity (**Figures 2B,C**). Our data validate results previously reported in other populations of *A. palmeri* (Gaines et al., 2010, 2011; Ribeiro et al., 2014), and other weedy plant species such as *A. tuberculatus* (Lorentz et al., 2014; Chatham et al., 2015), *Lolium perenne* ssp. *multiflorum* (Salas et al., 2012), *Eleusine indica* (Chen et al., 2015), and *Kochia scoparia* (Wiersma et al., 2015). Additionally our data confirmed the accumulation of shikimate following treatment with glyphosate, mostly in the GS population (**Figure 2D**). Shikimate is a known stress marker which accumulates following EPSPS inhibition in GS populations (Dyer et al., 1988; Baerson et al., 2002; Zhu et al., 2008; Whitaker et al., 2013; Doğramacı et al., 2015; Fernández-Escalada et al., 2016; Dillon et al., 2017).

### Gene Amplification of *EPSPS* in *A. palmeri* GR Populations Has No Major Pleiotropic Effect on the Expression of AAA Biosynthetic Genes

Despite all these traits that characterize a GR population at molecular and biochemical levels, our work revealed that gene amplification of *EPSPS* had no major effect on the overall AAA pathway (**Figures 2–5**). In particular, in untreated plants, the level of free AAA content was similar in GR and GS populations (**Figure 5**). Similar AAA content in glyphosate resistant/sensitive biotypes has been previously described (Maroli et al., 2015). This is consistent with previous reports suggesting that the overexpression of *EPSPS* may have no fitness cost in *A. palmeri* (Giacomini et al., 2014; Vila-Aiub et al., 2014).

The entrance of the primary metabolism to AAA pathway is through DAHPS enzyme (Tohge et al., 2013). Plants control the carbon flux into the pathway by controlling *DAHPS* transcription and protein abundance (Herrmann and Weaver, 1999). However, it was previously unknown whether GR populations with increased EPSPS expression would have altered *DAHPS* regulation. Higher levels of DAHPS activity were described in GR populations compared to sensitive populations in *Nicotiana tabacum L.* (Dyer et al., 1988) and *Convolvulus arvensis* (Westwood and Weller, 1997). In *Lolium rigidum GR* populations with higher *EPSPS* expression, levels of *DAHPS* transcripts were similar to sensitive population (Baerson et al., 2002). In this study, while *DAHPS* mRNA relative expression was similar in both populations (**Figure 3B**), the DAHPS protein level in GR was more than twofold higher than in GS (Supplementary Figures 1A,B). It could implicate a translational regulation (or at least post-transcriptional mechanism) that controls DAHPS, and this may be related to *EPSPS* gene overexpression.

### In Sensitive and Resistant Plants Glyphosate Treatment Provokes Increased Transcript Abundance Leading to Synthesis of Chorismate, and after This Regulatory Point, Tryptophan

Our study shows that glyphosate treatment provoked an accumulation of the transcripts encoding virtually all the enzymes of the shikimate pathway, including *EPSPS*, in a dose-dependent manner (**Figure 3B**). This trend seems to be specific for enzymes of the AAA pathway and was not observed for the enzymes of the BCAA pathway (**Figure 6**). Although increases in some enzymes of the shikimate pathway such as EPSPS (Baerson et al., 2002; Yuan et al., 2002; Chen et al., 2015; Mao et al., 2016) and DAHPS (Baerson et al., 2002) have been previously described, this is the first report suggesting a potential coordinated transcriptional regulation of the shikimate pathway after glyphosate treatment. Because this regulation is observed in both GS and GR populations (**Figure 3B**), it suggests that this gene upregulation does not occur in response to the level of inhibition of EPSPS activity. Instead, it can be hypothesized that glyphosate itself, or indirectly, may affect plant amino acid metabolism, in addition to its known impact on EPSPS. Future research is needed to determine if glyphosate has unreported effects on plants and what signal causes this general gene induction of the pre-chorismate pathway.

This general upregulation of the expression of genes participating in the pre-chorismate pathway is accompanied with an increase of the accumulation of free AAAs, which is more pronounced in the GS population (**Figure 5**). Although already reported (Vivancos et al., 2011; Maroli et al., 2015; Fernández-Escalada et al., 2016), this might appear counter-intuitive at first glance because glyphosate is inhibiting the entry of carbon in this biosynthetic pathway, and therefore is expected to prevent synthesis of AAA. It is possible that the accumulation of free AAA comes from an increase in protein turnover in the plant following glyphosate treatment (Zabalza et al., 2006; Zulet et al., 2013a; Fernández-Escalada et al., 2016). Isotopic studies in *A. palmeri* revealed that both *de novo* synthesis of amino acids and protein turnover contribute to AAA accumulation in response to glyphosate (Maroli et al., 2016). While gene expression induction after glyphosate was similar in GR and GS populations (**Figure 3B**), the accumulation of AAA was mainly observed in GS plants (**Figure 5**). That observation may suggest that AAA accumulation following glyphosate treatment is rather related to the level of stress experienced by the plant.

After chorismate, *AS* increase in transcript abundance was higher than any other enzyme in the pathway in response to glyphosate treatment (**Figure 3B**). *AS* expression was induced while *CM* expression was repressed, suggesting a regulatory branch point in the pathway (**Figure 1**) for a preferential flux of carbon toward Trp biosynthesis over Phe and Tyr biosynthesis. This potential stream toward Trp was confirmed by studying AS and CM enzyme activities *in vitro* (**Figure 4**). Data obtained in *Arabidopsis thaliana* (Sasaki-Sekimoto et al., 2005) and other plant species (Galili et al., 2016) also support this hypothesis. However, measurements of free AAA in treated plants did not reveal any specific accumulation of Trp. Instead all three AAA were accumulated to a similar extent in GS plants (**Figure 5**). Yet, a slight difference was detected in the GR plants, which may suggest that under “mild” stress (3x dose in GR), synthesis of Trp is prioritized over the synthesis of Phe and Tyr. It is possible that this regulation is related to the inhibition of DAHPS by arogenate (Siehl, 1997), an intermediate product of the CM pathway. DAHPS may be key to the regulation of shikimate synthesis because it represents the entry point in this pathway (Maeda and Dudareva, 2012). Interestingly, *DAHPS* gene expression was induced by glyphosate in both populations (**Figure 3B**) while the increase in DAHPS protein was only detected in GS population (Supplementary Figures 1A,B). This might indicate that other layers of regulation (post-transcriptional) might fine-tune the regulation of this pathway. PAL protein level and enzyme activity have also been studied, because it represents the most important output from the AAA pathway (Hahlbrock and Scheel, 1989). No differences were found between populations for PAL protein abundance or activity level in untreated and treated plants (Supplementary Figures 1C–E). While other studies with other species show important effects of glyphosate on PAL (Hoagland et al., 1979; Zabalza et al., 2017), our results show that PAL abundance and enzyme activity are not affected in *A. palmeri*.

The results obtained after glyphosate treatment suggest that a stress-induced response to glyphosate increases the enzyme expression in the AAA pathway, which may require a substantial increase in energy consumption (Benevenuto et al., 2017). Trying to increase the carbon flux, which could further increase shikimate accumulation upon glyphosate treatment, could lead to the loss of feedback control in the pathway (Marchiosi et al., 2009). Reduction in AAA levels does not appear to elicit the increased expression of AAA pathway genes, because the AAA concentrations increase with glyphosate dose. Further research is needed to understand the signal(s) that upregulates the AAA pathway following glyphosate treatment.

### No Cross Regulation between AAA and BCAA Pathway Was Detected

In general, the free amino acid pool increases after glyphosate treatment (Orcaray et al., 2010; Vivancos et al., 2011; Zulet et al., 2013a, 2015; Liu et al., 2015) but the higher relative increase is in BCAA levels (Orcaray et al., 2010). The higher effect of glyphosate on BCAA than on other amino acid types suggests a possible effect of the herbicide on the BCAA biosynthetic pathway. The expression pattern of the BCAA biosynthetic pathway was measured (**Figure 6**) and no clear patterns for expression changes of the BCAA enzymes in plants treated with glyphosate were identified (**Figure 6**), while an induction of expression of AAA enzymes was detected (**Figure 3B**). Although some authors (Guyer et al., 1995; Noctor et al., 2002; Pratelli and Pilot, 2014) have proposed cross-regulation between the levels of AAA and BCAA, and close correlation was observed between the AAA pathway and the BCAA pathway (Noctor et al., 2002), no cross-regulation at the transcriptional level was found in this study.

## Conclusion

No differences were found (other than *EPSPS*) in transcriptional regulation of the shikimate pathway between *A. palmeri* GR and GS untreated plant, which implies that pleiotropic effects due to shikimate pathway perturbation are not apparent. Transcriptional induction of the AAA pathway was detected following glyphosate treatment in both GR and GS plants, suggesting a potential coordinated transcriptional regulation. AAA content was not the signal causing this response, because AAA accumulation was detected only in GS plants and further research will be needed to determine the signal. Glyphosate treatment resulted in an upregulation of the Trp biosynthesis branch instead of the Phe and Tyr branch, indicating that this branch point may be a regulatory point in the pathway. With respect to cross-regulation between the AAA and BCAA pathways, no differences in BCAA transcriptional regulation were found due to either EPSPS gene amplification or to glyphosate treatment.

## Author Contributions

MR and AZ conceived and designed the experiments, performed by MF-E; KR and MG-M contributed with analysis tools; MF-E, AZ-G, and MG-M prepared figures; MF-E, AZ, KR, TG, and MR analyzed, discussed results and wrote the manuscript. All authors read and approved the final manuscript.

## Conflict of Interest Statement

The authors declare that the research was conducted in the absence of any commercial or financial relationships that could be construed as a potential conflict of interest.
