# Supplementary material for: Effects of EPSPS Copy Number Variation (CNV) and Glyphosate Application on the Aromatic and Branched Chain Amino Acid Synthesis Pathways in Amaranthus palmeri
Source: Front Plant Sci. 2017 Nov 16;8:1970. doi: 10.3389/fpls.2017.01970 (PMC5696356; doi:10.3389/fpls.2017.01970)
Supplement: Supplementary file 1 [file Table_1.DOCX]

**Table 1.** Primers (5’-3’) used in the quantitative RT-PCRs.

| **GENE** | **FORWARD** | **REVERSE** | **Annealing temperature** | **Efficiency** |
| --- | --- | --- | --- | --- |
| *Aromatic amino acid biosynthetic pathway* | | | |  |
| DAHPS | cctcataggatgataagggc | ctttgcatggcagcataacc | 55 ºC | 96 % |
| DHQS | gcattgttggctagggatcc | aacctcggccttgttttcac | 61 ºC | 91 % |
| DQSD | ggtgtactcaagcaaggagc | tgtggactcttactatggcc | 57 ºC | 84 % |
| SK | gattctgaagcacaaagcagc | cagttgttttcccagagccc | 55 ºC | 91 % |
| EPSPS | aatgctaaaggaggccttcc | tcaatctccacgtctccaag | 61 ºC | 93 % |
| CS | cttgatagaaggaggcctgg | gtttctttcctaggagtagtg | 57 ºC | 90 % |
| CM | gaatacattatggcaagtatgt | gtcataagtcgctccttgtc | 52ºC | 97 % |
| AS | tttggagggaaggttgtgcg | ctggtgagctttttccatgc | 57 ºC | 88 % |
| *Branched chain amino acid biosynthetic pathway* | | | |  |
| AHAS | cttcctcgacatgaacaagg | attagtagcacctggacccg | 57 ºC | 84 % |
| AHAIR | atggctcagattgagatcttg | ccacggcttcaatcacactc | 52 ºC | 90 % |
| DHAD | taccatggcatcagctatcg | ggtgttgacgagctgtaagg | 55 ºC | 96 % |
| TA | gtgaagatgatcttcgtcggc | tcacaatcagacttgaaagatg | 52 ºC | 99 % |
| *Normalization gene* | |  |  |  |
| Beta tubulin | gatgccaagaacatgatgtg | tccacaaagtaggaagagttc | 55 ºC | 90 % |
